# Serum neurofilament light chain as a biomarker of disease control in multiple sclerosis: a real-world cross-sectional analysis of therapeutic regimens

**DOI:** 10.1007/s00415-025-13573-4

**Published:** 2025-12-19

**Authors:** Agni M. Konitsioti, Finja Schweitzer, Wibke Johannis, Falk Steffen, Stefan Bittner, Gereon R. Fink, Michael Schroeter, Clemens Warnke

**Affiliations:** 1 Department of Neurology, Faculty of Medicine and University Hospital Cologne, University of Cologne, Kerpener Str. 62, 50937 Cologne, Germany; 2 Institute for Clinical Chemistry, Faculty of Medicine and University Hospital Cologne, Cologne, Germany; 3 Department of Neurology, Focus Program Translational Neuroscience (FTN) and Immunotherapy (FZI), Rhine Main Neuroscience Network (Rmn(2)), University Medical Center of the Johannes Gutenberg University Mainz, Mainz, Germany; 4 Cognitive Neuroscience, Institute of Neuroscience and Medicine (INM3), Research Center Jülich, Jülich, Germany; 5 Department of Neurology, University Hospital Marburg, Marburg, Germany

**Keywords:** Multiple Sclerosis, biomarker, extended intervall, sNfL, immunotherapy, therapeutic regimens

## Abstract

**Background:**

Serum neurofilament light chain (sNfL) is an established biomarker of disease activity and progression in persons with multiple sclerosis (PwMS), with studies showing elevated sNfL levels during relapses and positive associations with disability scores.

**Objective:**

To assess sNfL levels in PwMS receiving different disease-modifying therapies (DMTs), with a particular focus on extended-interval dosing (EID) regimens in real-world clinical practice.

**Methods:**

In this two-center cross-sectional study, 172 PwMS without relapses in the preceding three months were included (University Hospital Cologne, n = 125; University Hospital Mainz, n = 47). Patients were categorized into the following groups: (1) low-efficacy DMT (leDMT; n = 8), (2) natalizumab standard-interval dosing (SID; every 4 weeks; n = 7), (3) natalizumab EID (every 6–8 weeks; n = 53), (4) ofatumumab (n = 17), (5) ocrelizumab SID (every 6 months; n = 48), (6) ocrelizumab EID (every 9 months; n = 17), and (7) no DMT (n = 19). sNfL levels were measured once in a cross-sectional design using an electrochemiluminescence immunoassay.

**Results:**

No significant differences in sNfL levels were observed across DMT subgroups in the ANCOVA analysis after adjusting for age and the presence of new T2 lesions on the most recent cranial MRI. However, PwMS receiving DMTs showed lower sNfL levels compared with untreated patients. Notably EID of ocrelizumab (every 9 months; 1.56 pg/mL, 95% CI 1.26–1.85) and natalizumab (every 8 weeks; 1.46 pg/mL, 95% CI 1.29–1.64) was not associated with higher sNfL levels compared to standard interval dosing (SID) of ocrelizumab (1.45 pg/mL, 95% CI 1.27–1.63) or natalizumab (1.13 pg/mL, 95% CI 0.68–1.58).

**Conclusion:**

EID regimens were not associated with increased sNfL levels, suggesting that they may effectively limit neuroaxonal damage. Larger studies that assess the added value sNfL monitoring for safely personalizing treatment intervals in PwMS with initially active disease are needed.

**Supplementary Information:**

The online version contains supplementary material available at 10.1007/s00415-025-13573-4.

## Introduction

Multiple sclerosis (MS) is a chronic, immune-mediated inflammatory demyelinating disease of the central nervous system (CNS) affecting over 2 million individuals worldwide [1]. Permanent disability arises from focal inflammation, diffuse neuronal damage, and impaired repair mechanisms [2]. Oligospecific intrathecal immunoglobulin synthesis, demonstrated by CSF-restricted IgG oligoclonal bands (OCB) or an increased free kappa light chain index, represents a hallmark of MS [3, 4]. In addition to assisting in diagnosing MS, blood biomarkers reflecting tissue damage and enabling subclinical activity monitoring may help to evaluate therapeutic responses, individualize treatment regimens, and predict disability in MS [5]. Neurofilament proteins are among the most extensively studied blood-based biomarkers for neuronal injury and loss across various diseases, including MS [6]. The development of high-sensitivity assays, such as the single-molecule array (SIMOA) technology [7], has enabled the detection of serum Nfl (sNfL) at single-digit picogram/milliliter concentrations, allowing for minimally invasive, longitudinal monitoring of sNfL levels. These advances have paved the way for sNfL integration into clinical practice as a biomarker for disease activity and progression in MS.

Numerous studies have demonstrated that both CSF NfL and sNfL levels are elevated during relapses in MS [8, 9] and positively associated with disability scores [9–11] and magnetic resonance imaging (MRI)-based measures of inflammatory disease activity [12–14]. sNfL has been used as a marker of treatment response [15, 16], serving as an efficacy endpoint in trials of treatments for RRMS and secondary progressive MS (SPMS) [17–20]. A more pronounced decrease in sNfL levels of MS patients treated with high-efficacy disease-modifying therapies (DMT) such as CD20, CD52, and α4β1-integrin monoclonal antibodies, compared to oral therapies (S1P receptor modulators, dimethyl fumarate, teriflunomide) or platform therapies (glatiramer acetate, IFNβ) has been shown [20–24].

Neuroaxonal damage in MS is driven both by inflammatory disease activity and chronic neurodegeneration. sNfL may thus be understood as an integrator over these components, reflecting the compound neuroaxonal damage. While transient spikes of sNfL may indicate recent (sub-) acute inflammatory activity, sustained elevations may suggest chronic ongoing neuroaxonal damage driving progression [25–27]. Additionally, sNfL could serve as an added tool in clinical practice to monitor inflammatory disease activity in MS during immune therapy [28], being particularly valuable in cases of “clinically silent disease,” or in situations where differentiating between relapses and pseudo-relapses is challenging [29].

Studying sNfL in real world settings captures patient heterogeneity, treatment concepts that deviate from product information, comorbidities, and variable disease courses, often not reflected in pivotal clinical trials. Albeit the formal evidence level is lower, real-world findings may provide added practical information for physicians and their patients facilitating clinical decision-making, including personalized treatment adjustments, bridging the gap between trial findings and routine clinical practice [30, 31].

Natalizumab, typically administered at a standard interval dosing of 300 mg every 4 weeks (SID), is effective but carries a risk of progressive multifocal leukoencephalopathy (PML), a potentially fatal condition caused by the JC virus [32]. SID maintains natalizumab concentrations at levels that ensure 70–80% continuous α4β1 integrin receptor saturation [33]. However, studies have shown that lower receptor occupancy can effectively block autoreactive immune cell extravasation, which is responsible for CNS attacks in RRMS [34, 35]. In non-randomized observational studies, extending the interval between natalizumab doses to 6 weeks (in one study up to 7 weeks) has been associated with a significantly lower risk of PML compared to SID [36–39] while showing similar efficacy to SID in terms of relapse rate, Expanded Disability Status Scale (EDSS), MRI lesions, and sNfL levels [37, 38, 40–42]. However, interruptions longer than 12 weeks in NZ treatment led to increased risk of disease activity [35, 43–45]. Also, in one study of patients receiving natalizumab every 6 weeks, a significant increase in the proportion of patients complaining of wearing-off was reported [46].

Similarly, ocrelizumab’s SID consists of an induction phase (two 300 mg infusions 14 days apart) followed by a maintenance phase of 600 mg every 6 months. Recent evidence has shown that B-cell depletion starts 2 weeks after infusion and can last for more than 6 months [47]. During the COVID-19 pandemic, the postponed administration of ocrelizumab allowed for the assessment of extended interval dosing (EID) effects. Results have shown that extending ocrelizumab dosing up to 9.9 months maintains treatment efficacy, as evaluated by relapse rates, MRI activity, and disability progression in both RRMS and primary progressive MS (PPMS) patients [48–52]. Furthermore, ocrelizumab EID has been shown to maintain stable levels of IgG, IgM, and IgA, or to result in lower rates of hypo-IgM (< 40 mg/dL) [53, 54], though one study indicated that EID may be associated with lower rates of B-cell depletion [55].

## Patients and methods

This two-center, cross-sectional study included 125 MS patients with stable disease undergoing immunotherapy at the University Hospital Cologne between April and September 2024, and 47 MS patients at the University Hospital Mainz (i.e., the total cohort comprised of 172 patients). Inclusion criteria were: age 18 years or older, a diagnosis of RRMS, PPMS, or SPMS, at least 2 years of immunotherapy treatment, and a stable disease with no relapses in the preceding 3 months. Stable disease was defined in relation to sNfL levels as no relapses within the preceding 3 months, in line with findings indicating that sNfL concentrations generally return toward baseline within approximately 3 months following an MS relapse [56]. Recruitment and serum sample collection happened between April and September 2024. We collected demographic, clinical, and radiological data from clinical routine. Blood sampling of patients receiving Natalizumab or Ocrelizumab was performed upon establishing intravenous access, directly before administration of the next dose. Sampling of all other patients took place during routine follow-up visits in the outpatient clinic. The study was approved by the Ethics Committee of the University Hospital of Cologne (protocol Nr. 18-266), and written informed consent was obtained from all participants prior to their inclusion. Notably, DMT had been selected, and dosing intervals had been adapted as per the decision of the treating physician before study inclusion. DMT were categorized into lower-efficacy DMT (leDMT, including interferons, fumarates, glatiramer acetate, and teriflunomide, N = 8), S1P modulators or cladribine (N = 3), natalizumab standard interval dosing (SID, every 4 weeks, N = 7), natalizumab extended interval dosing (EID, every 6–8 weeks, N = 53), ofatumumab (N = 17), ocrelizumab SID (every 6 months, N = 48), ocrelizumab EID (every 9 months, N = 17), and no DMT (N = 19).

Serum samples were collected once in a cross-sectional design to assess sNfL levels, following current evidence that supports the use of serum over plasma for NfL measurement in large-scale clinical laboratories [23]. For pre-processing of samples, approximately 3.0 mL of blood was collected into serum separator tubes. Samples were allowed to clot 30–60 min at room temperature before centrifugation with a swing bucket for 10 min at 1200 G-force or 15 min in a fixed angle centrifuge. Serum samples were stored at − 80 °C at the University Hospital of Cologne or University Hospital of Mainz until processed. The Elecsys NfL assay (Roche Diagnostics), an electrochemiluminescence immunoassay (ECLIA) utilizing a research-only kit, was used on a Roche cobas e 801 analyzer for quantitative and standardized in-vitro detection of sNfL.

Statistical analyses and graphical representations were performed using SPSS (Version 30.0). Descriptive statistics were applied to demographic and clinical variables, with categorical variables expressed as counts and percentages, and continuous or ordinal variables presented as medians with interquartile ranges (IQRs). For all analyses, sNfL concentrations were right-skewed with outliers and heavy-tailed. They were analyzed after log transformation to meet the assumption of a normal distribution of the residuals required in regression models, with median values as summary statistics as previously described [57]. An example of the distribution of sNfL values, shown as histograms before and after log₁₀ transformation, is presented in Supplementary Fig. 1. The level of statistical significance for all tests was set at p = 0.05.

To estimate the median sNfL and its associated uncertainty, we applied a non-parametric single-variable bootstrapping approach. The input consisted of sNfL values from all 120 patients. The dataset was resampled with replacement to generate 1,000 bootstrap samples, each containing the same number of patients as the original cohort. For each bootstrap sample, the median sNfL was calculated, producing an empirical distribution of median values. The output of this procedure included the bootstrap median as the point estimate, the 95% confidence interval derived from the 2.5th and 97.5th percentiles, and the interquartile range (IQR) of the bootstrap medians as an additional measure of variability. This approach allowed robust estimation of median sNfL and its uncertainty while accounting for the variability in the original dataset.

Linear univariate and multivariate regression models were employed to examine associations with log-transformed sNfL. Given the substantial age-dependent increase in sNfL levels, adjustment for age and other confounding factors was included in the regression models, though non-linearity presented challenges. Kruskal–Wallis tests were used for inter-group comparisons of sNfL values. To compare sNfL levels across different therapeutic regimens, an Analysis of Covariance (ANCOVA) was applied, allowing for statistical control of the covariates with a definite effect on sNfL, thereby improving precision and removing a potential source of bias.

## Results

sNfL measured using the Elecsys ECLIA assay has been shown to correlate strongly with Simoa levels and is a reliable method for assessing sNfL in MS [31]. Most published MS cohorts report sNfL using the SiMoA method, with median concentrations of 7–20 pg/mL depending on disease stage and activity [58]. However, as ECLIA systematically yields lower values than SiMoA [59], direct numeric comparisons were not appropriate in this study. Consequently, our analyses focus on within-cohort relationships rather than comparisons of absolute sNfL values to other studies. As all sNfL measurements were performed using the ECLIA method in a single laboratory, ensuring consistency and reliability, we chose to use—instead of z-scores—raw sNfL values, and include age as a covariate in further analyses. Moreover, since relatively few studies report sNfL values obtained via ECLIA, we believe that our data provide valuable additional information to the existing literature.

### Patient demographics and sNfL levels

The cohort consisted of 172 patients, predominantly female (n = 70, 59%). The median age was 42.0 years (IQR = 31.0–52.0). The median sNfL concentration was 1.29 pg/mL, as measured by ECLIA (IQR = 0.96–1.83). The median treatment duration was 3.24 years (IQR = 2.00–6.00), the median EDSS score was 3.0 (IQR = 1.0–4.0), and the median disease duration was 7.0 years (IQR = 4.0–14.0). Among the cohort, 138 patients (81.2%) had RRMS, 16 (9.4%) had PPMS, and 16 (9.4%) had secondary SPMS. MRI data were derived from written radiological reports provided either by in-house radiologists or by external radiology practices where patients underwent their routine MRIs. Brain MRI data were available for 103 patients (Table 1), and patients with missing MRI data (n = 18) were excluded from MRI-related analyses.

**Table 1:** Descriptive statistics for the overall cohort, including the number and percentage of patients, as well as corresponding sNfL levels, across subgroups defined by sex, age, MS course, treatment regimen, MRI findings, and occurrence of relapse within the past 6 months

Variable	N	%	sNfL (pg/mL), median (IQR)
Age			42.0 (31.0–52.0)
NfL (pg/mL) (ECLIA)			1.29 (0.96–1.83)
Years under treatment			3.24 (2.00–6.00)
EDSS			3.0 (1.0–4.0)
Disease duration			7.0 (4.0–14.0)
*Sex*			
Male	102	41	1.34 (1.06–1.89)
Female	70	59	1.23 (0.92–1.76)
*Age group (years)*			
< 30	38	22.1	0.67 (0.76–1.17)
30–40	40	23.3	1.08 (0.89–1.35)
40–50	46	26.7	1.37 (1.07–1.72)
50–60	31	18.0	1.89 (1.29–2.35)
> 60	17	9.9	2.27 (1.85–2.98)
*MS course*			
RRMS	138	81.2	1.23 (0.90–1.72)
PPMS	16	9.4	1.83 (1.37–1.98)
SPMS	16	9.4	1.76 (1.05–2.64)
*Treatment*			
leDMT	8	4.7	1.20 (0.93–1.77)
Ocrelizumab SID (every 6 months)	48	27.9	1.34 (1.02–1.82)
Ocrelizumab EID (every 9 months)	17	9.9	1.61 (0.96–2.35)
Ofatumumab	17	9.9	1.25 (1.00–1.49)
Natalizumab SID	7	4.1	1.03 (0.50–1.21)
Natalizumab EID (every 6–8 weeks)	53	30.8	1.12 (0.87–1.49)
S1P inhhibitor or cladribin	3	1.7	1.06 (1.06–1.06)
noDMT	19	11	2.73 (1.83–3.11)
*New T2 lesions in last cranial MRI*^a^			
Yes	15	14.0	2.42 (1.06–2.91)
No	92	86.0	1.28 (1.00–1.87)
*Relapse in the past 6 months*			
Yes	21	12.3	1.39 (0.97–2.28)
No	150	87.7	1.30 (0.96–1.81)

*sNfL* serum neurofilament light chain (protein), *IQR* interquartile ranges, *MS* multiple sclerosis, *EDSS* Expanded Disability Status Scale, *DMT* disease modifying therapy, *leDMT* lower efficacy disease modifying therapy, *SID* standard interval dosing, *EID* extended interval dosing, *RRMS* relapsing–remitting MS, *SPMS* secondary progressive MS, *PPMS* primary progressive MS, *MRI* magnetic resonance imaging

^a^Conducted within the last 12 months

Comparison of sNfL levels in patients with new T2 lesions on their most recent MRI (performed within the past 12 months) revealed significantly higher sNfL concentrations in these patients (median 2.42 pg/mL, IQR 1.06–2.91) compared to those without new lesions (median 1.28 pg/mL, IQR 1.00–1.87; Kruskal–Wallis test p = 0.029). Comparison of sNfL levels among patients with different MS forms (RRMS, PPMS, and SPMS) revealed significant differences (Kruskal–Wallis test, p = 0.008). Post-hoc analysis indicated significantly lower sNfL levels in patients with RRMS (median 1.23 pg/mL, IQR 0.90–1.72) compared to those with PPMS (median 1.83 pg/mL, IQR 1.37–1.98; p = 0.034). However, after adjusting for age in the ANCOVA, these differences were no longer statistically significant. Given the substantial imbalance in group sizes (RRMS, n = 138 vs. PPMS, n = 16), the data do not allow definitive conclusions (Supplementary Table 1A and B).

**Fig. 1:**
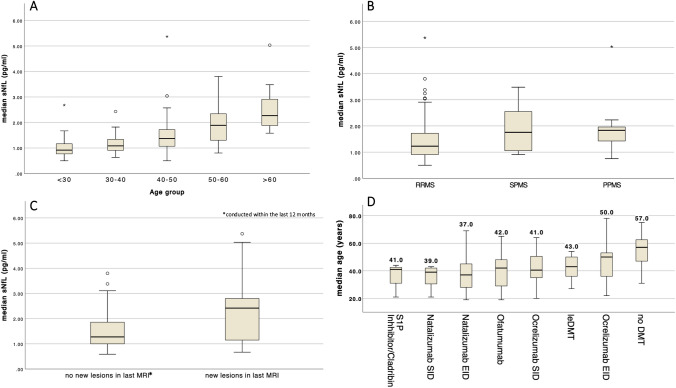
Descriptive statistics Legend Figure 1: **A**–**C**, sNfL concentrations across different groups in our cohort (age, MS course, and MRI findings from the most recent MRI conducted within the last 12 months). **D**, median age (in years) across different treatment groups: leDMT (N = 8), S1P modulators or cladribine (N = 3), natalizumab SID (N = 7), natalizumab EID (N = 53), ofatumumab (N = 17), ocrelizumab SID (N = 48), ocrelizumab EID (N = 17), and no DMT (N = 19). MS, multiple sclerosis; DMT, disease-modifying therapy; leDMT, lower efficacy disease-modifying therapy; SID, standard interval dosing; EID, extended interval dosing; sNfL, serum neurofilament light chain (protein); RRMS, relapsing–remitting MS; SPMS, secondary progressive MS; PPMS, primary progressive MS; MRI, magnetic resonance imaging

Significant age differences were observed between therapy groups, with the noDMT group being older (median 57 years) compared to the groups receiving leDMT (43 years), ocrelizumab SID (41 years), ocrelizumab EID (50 years), natalizumab SID (39 years), natalizumab EID (37 years), and ofatumumab (42 years). Additionally, patients in the ocrelizumab EID group (median age 50 years) were significantly older than those in the ocrelizumab SID (41 years), natalizumab SID (39 years), and natalizumab EID (37 years) groups (Fig. 1D). Table 1 provides the descriptive statistics, and Fig. 1 provides a graphical representation of the demographic data.

Several variables were tested for their association with sNfL levels in the entire cohort using both univariate and multivariate regression models. In the univariate regression model, a positive association was found between sNfL and age (b = 0.593, 95% CI 0.030–0.046, p < 0.0001), disease duration (b = 0.205, 95% CI 0.005–0.036, p = 0.008), EDSS (b = 0.335, 95% CI 0.073–0.186, p = < 0.001), new T2 lesions on the most recent cranial MRI (b = 0.352, 95% CI 0.430–1.387, p = < 0.001), presence of relapses within 6 months before sampling (b = 0.168, 95% CI 0.038–0.018, p = 0.032), treatment status (treated vs. untreated; b = − 0.491, 95% CI − 1.559 to − 0.887, p < 0.001), and disease course (RRMS vs PPMS/SPMS, b = 0.207, 95% CI 0.111–0.729, p = 0.008). No significant association was observed between sNfL and treatment duration in years (b = 0.068, 95% CI − 0.018 to 0.044, p = 0.419) or gender (b = 0.050, 95% CI − 1.169 to 0.332, p = 0.521) (Table 2).

**Table 2:** Associations between sNfL and demographic/clinical variables using univariate and multivariate regression models

Variable	Univariate regression sNFL					Multivariate regression sNFL				
	R squared	b*	95% CI		p	R squared	b*	95% CI		p
			Lower	Upper				Lower	Upper	
Age	0.352	0.593	0.030	0.046	**< 0.001**	0.525	0.510	0.022	0.050	**< 0.001**
Disease duration	0.042	0.205	0.005	0.036	**0.008**	0.525	− 0.151	− 0.034	0.003	0.096
Years under treatment	0.005	0.068	− 0.018	0.044	0.419	–	–	–	–	–
EDSS	0.112	0.335	0.073	0.186	**< 0.001**	0.525	0.109	− 0.032	0.130	0.233
New T2 lesions in last cranial MRI	0.124	0.352	0.430	1.387	**< 0.001**	0.525	0.255	0.208	1.105	**0.005**
Relapse in the last 6 months	0.028	0.168	0.038	0.818	**0.032**	0.525	− 0.006	− 0.390	0.362	0.939
Gender	0.003	0.050	− 1.169	0.332	0.521	–	–	–	–	–
Treated vs. Untreated^a^	0.241	− 0.491	− 1.559	− 0.887	**< 0.001**	0.525	− 0.262	− 1.108	− 0.140	**0.012**
Disease course (RRMS vs. PPMS/SPMS)	0.043	0.207	0.111	0.729	**0.008**	0.525	− 0.041	− 0.554	0.342	0.639

Statistically significant results are indicated in bold

b* b standardized, *CI* confidence interval, *EDSS* Expanded Disability Status Scale, *MS* multiple sclerosis, *DMT* disease-modifying therapy, *leDMT* lower efficacy disease-modifying therapy, *sNfL* serum neurofilament light chain (protein), *RRMS* relapsing–remitting MS, *SPMS* secondary progressive MS, *PPMS* primary progressive MS, MRI magnetic resonance imaging

^a^Among these, 4 patients were newly diagnosed (but have not experienced any relapses in the last three months), while the remaining patients have not received any DMT for several years.

In the multivariate model, the following variables that showed a statistically significant positive association in the univariate analyses were included: age, disease duration, EDSS, new T2 lesions on the most recent cranial MRI, presence of relapses within 6 months before sampling, DMT treatment status (treated vs. untreated), and disease course (RRMS vs PPMS/SPMS. After adjustment, sNfL levels remained significantly associated with age (b = 0.510, 95% CI 0.022–0.050, p = < 0.001), new T2 lesions on the most recent cranial MRI (b = 0.255, 95% CI 0.208–1.105, p = 0.005), and DMT treatment status (b = − 0.262, 95% CI − 1.108 to − 0.140, p = 0.012). The association between disease duration, EDSS, presence of relapses within 6 months before sampling, disease course, and sNfL was no longer significant in the multivariate model (Table 2).

### Comparison of sNfL levels across DMT regimens

To compare sNfL levels across different therapeutic regimens, an Analysis of Covariance (ANCOVA) was conducted, adjusting for age and presence of new T2 lesions in the last cranial MRI as covariates. Group receiving S1P inhibitors/cladribin were excluded from the analysis due to small sample sizes.

**Fig. 2:**
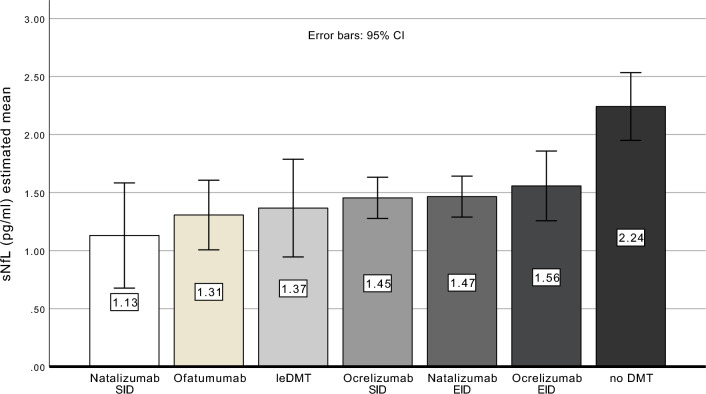
Estimated mean sNfL values (in pg/mL) after ANCOVA analysis across the different treatment groups using age and presence of new T2 lesions in the last cranial MRI as covariates Legend Figure 2: ANCOVA: analysis of covariance, CI: confidence interval, sNfL: serum neurofilament light chain (protein), SID: standard interval dosing, EID: extended interval dosing, leDMT: lower efficacy disease-modifying therapy

The following trends were noted: patients in the Natalizumab SID group had the lowest sNfL levels (1.13 pg/mL), followed by Ofatumumab (1.31 pg/mL), leDMT (1.36 pg/mL), Ocrelizumab SID (1.45 pg/mL), and Natalizumab EID (1.46 pg/mL). Ocrelizumab EID showed slightly higher levels (1.56 pg/mL), whereas untreated patients (no DMT) had the highest sNfL concentrations (2.24 pg/mL). The noDMT group exhibited significantly higher sNfL levels than all DMT-treated groups, while differences among the DMT groups themselves did not reach statistical significance (Table 3A and B, Fig. 2).

**Table 3:** (A) ANCOVA results for the overall model when comparing sNfL levels across various therapeutic regimens. (B) Post-hoc analysis of sNfL differences between therapy groups

(A) ANCOVA				
	Mean square	F	p	Partial eta squared
*Leven’s test of equality of error variances*			< 0.001	
*Variable*				
Treatment	1.749	4.83	< 0.001	0.159
*Covariates*				
Age	16.1	44.56	< 0.001	0.024
Presence of new T2 lesions in last cranial MRI	1.34	3.72	0.055	0.224

(B) Post-hoc analysis				
Treatment	Mean estimated sNfL (pg/mL)	95% CI		p
		Lower	Upper	
leDMT	1.36	0.94	1.78	vs. noDMT: *p* = 0.020
Ocrelizumab SID	1.45	1.27	1.63	vs. noDMT: * p* = < 0.001
Ocrelizumab EID	1.56	1.26	1.85	vs. noDMT: * p* = 0.025
Ofatumumab	1.31	1.01	1.61	vs. noDMT: * p* = < 0.001
Natalizumab SID	1.13	0.68	1.58	vs. noDMT: * p* = 0.002
Natalizumab EID	1.46	1.29	1.64	vs. noDMT: * p* = < 0.001
noDMT	2.24	1.95	2.53	–

*ANCOVA* analysis of covariance, *CI* confidence interval, *sNfL* serum neurofilament light chain (protein), *SID* standard interval dosing, *EID* extended interval dosing, *RRMS* relapsing–remitting MS, *SPMS* secondary progressive MS, *PPMS* primary progressive MS, *EDSS* Expanded Disability Status Scale, *leDMT* low-efficacy disease-modifying therapy

## Discussion

sNfL has emerged as a biomarker of neuroaxonal damage, facilitating monitoring of disease activity, treatment response, and prognostication of disease progression in MS patients at the group level. Accurate prognostication at the individual level remains challenging due to inter-individual variability in disease activity and progression, differences in treatment response, and the lack of validated biomarkers.

In a multivariate regression model, we identified that age, presence of new T2-weighted lesions on the most recent MRI, and treatment status were independently associated with sNfL levels. Additionally, patients with PPMS and SPMS exhibited significantly higher sNfL levels compared to those with RRMS; however, this was not the case when adjusting for age.

Our findings corroborate previous research demonstrating a positive correlation between sNfL levels and the occurrence of new T2-weighted lesions [60] as well as with age, likely reflecting age-related neuronal degeneration [61]. The observed association with age may also indicate that disease progression in later stages reflects both direct neuronal damage and reduced or exhausted compensatory mechanisms. In our cohort, no significant difference in sNfL levels was observed between genders, which aligns with findings from other studies [61]. The association between sNfL levels and disability, as measured by the EDSS, suggesting a link between sNfL and both acute inflammatory damage and chronic diffuse neurodegeneration contributing to disability progression, was observed in the univariate analysis but did not remain significant in the multivariate model.

When analyzing sNfL levels across different DMT regimens, we detected no statistically significant differences between treatment groups. In contrast, we observed significantly higher sNfL levels without DMT (as compared with those treated with various DMTs). These findings are consistent with previous studies, suggesting that DMTs reduce sNfL levels, thereby underscoring their potential as biomarkers for monitoring treatment response [61]. Notably, ofatumumab was associated with sNfL levels comparable to the low levels observed with intravenous monoclonal antibody therapies. This finding is a critical observation, as previous comparative data on ofatumumab’s effect on sNfL levels were limited to the phase 3 ASCLEPIOS I/II trials that demonstrated superior efficacy outcomes for ofatumumab compared to teriflunomide [18]. Although it could be argued that patients receiving ofatumumab, as a DMT approved not before 2021, are younger and thus exhibit lower sNFL levels, age did not differ significantly between ofatumumab and the other DMT groups in our study (Fig. 1D).

As part of a clinical routine decision by the treating physician, natalizumab and ocrelizumab were used with SID and EID (every 9 months for ocrelizumab, every 6–8 weeks for natalizumab) in our cohort. Different dosing interval regimens showed comparable sNfL levels across treatment groups, and EID did not correlate with higher sNfL levels. Only a limited number of studies have explored natalizumab EID intervals longer than 7 weeks, with findings indicating no significant difference in relapse rates between EID and SID [37, 42]. Similarly, consistent with our results, most of the available studies highlighted no difference in sNfL levels between ocrelizumab SID and EID [54].

EID regimens may enhance vaccination responses to novel pathogens while simultaneously reducing the risks of infections and the complications associated with continuous immunosuppression [62, 63]. An extended treatment-free period could also provide sufficient time for a drug-free pregnancy while still offering protection from disease activity.

The “hit hard and early” strategy favours starting immunotherapies with highly effective substances [64]. However, it lacks any recommendation on how and when to de-escalate, and evidence remains scarce. At present, extended dosing intervals are widely used as a step to de-escalate. Efforts to define a generalizable EID protocol are ongoing, and the potential for personalized dosing schedules based on biomarkers and individual pharmacokinetic responses may offer more effective dosing strategies for MS patients. The feasibility of such an approach has already been demonstrated in a study where monitoring natalizumab serum concentrations was used to determine the optimal EID period for maintaining efficacy [65]. Additionally, studies in rituximab-treated MS patients suggest that B-cell monitoring may allow for EID of B-cell depleting therapies without compromising effectiveness. By contrast, sNFL levels render information from the CNS, not the blood compartment.

## Limitations

The main limitation of our study is the relatively small and heterogeneous study population, which also did not allow us to account for comorbidities or vascular risk factors, thus preventing us from assessing potential effects of alternative causes and comorbidities on sNfL [20]. Furthermore, due to the relatively small sample size, we focused our analysis on group-level trends instead of individual prognostication. Regarding natalizumab EID, it has been established that body weight influences the degree of α4-integrin saturation, with the efficacy of natalizumab decreasing as both the dosing interval and body weight increase [66]. Similarly, for ocrelizumab, previous studies have identified body weight as a covariate affecting ocrelizumab serum concentrations over time [67]. Although body weight data were not available in our study, it may represent a helpful factor to consider in future research aimed at optimizing individualized treatment regimens, particularly for patients with lower body weight. An additional limitation to this study is the comparison of patients with different disease phenotypes. Therapeutic interventions were not uniformly distributed across these groups due to regulatory restrictions, but also due to a limited study size. As a result, observed differences between groups may be confounded, and direct comparisons should be interpreted with caution. Finally, as this is a cross-sectional study, long-term efficacy of EID could not be assessed.

## Conclusion

Our findings support the utility of sNfL as a clinically meaningful blood biomarker for monitoring therapeutic effects in MS. In our cohort of clinically stable patients treated with various high-efficacy monoclonal antibodies, sNfL levels were comparable across treatment groups. EID of ocrelizumab (every 9 months) and natalizumab (every 8 weeks) was not associated with increased sNfL levels, suggesting that such regimens may effectively limit neuroaxonal damage while likely reducing the risks associated with continuous immunosuppression. Given the potential advantages of biologically tailored dosing in terms of safety, larger studies incorporating immunophenotyping and the detection of subclinical disease activity are warranted to validate the use of EID combined with sNfL monitoring as a strategy for gradual treatment de-escalation in patients with previously active MS.

## Conflicts of interest

CW received institutional support or personal fees for lecturing from Novartis, Alexion, Sanofi Genzyme, Biogen, Merck, Janssen, Bayer, Roche and Juvisé. MS received institutional support or personal fees for lecturing from Alexion, Argenx, Biogen, Datamed, Diaplan, Grifols, Novartis, Roche, Sanofi, Simon&Kucher, and UCB. SB received honoraria from Biogen, Bristol Myers Squibb, Hexal, Merck Healthcare, Novartis, Roche, Roche Diagnostics, Sanofi and Teva. Further, SB’s research is supported by the Deutsche Forschungsgemeinschaft (DFG, SFB CRC TRR 355-480846870), Novartis, and the Hermann- and Lilly-Schilling Foundation. AK, FS, WJ, FS and GRF states that he has no conflicting interests to declare.

## Supplementary Information

Below is the link to the electronic supplementary material.

Supplementary file1 (DOCX 162 KB)

## Data Availability

Anonymized data will be made available on request for any qualified investigator under the terms of the registry’s usage and access guidelines and subject to the informed consent of the patients.

## Abbreviations

IQR: Interquartile ranges
MS: Multiple sclerosis
DMT: Disease modifying therapy
leDMT: Low efficacy disease modifying therapy
SID: Standard interval dosing
EID: Extended interval dosing
sNfL: Serum neurofilament light chain (protein)
RRMS: Relapsing–remitting MS
SPMS: Secondary progressive MS
PPMS: Primary progressive MS
MRI: Magnetic resonance imaging
ANCOVA: Analysis of covariance
EDSS: Expanded Disability Status Scale
PML: Progressive multifocal leukoencephalopathy
JC: John Cunningham
SIMOA: Single-molecule array
ECLIA: Electrochemiluminescence immunoassay
BMI: Body mass index
CNS: Central nervous system
CSF: Cerebrospinal fluid

## References

[1] Walton C, King R, Rechtman L et al (2020) Rising prevalence of multiple sclerosis worldwide: insights from the Atlas of MS, third edition. Mult Scler J 26:1816–1821. 10.1177/1352458520970841 10.1177/1352458520970841 PMC7720355 33174475

[2] Lassmann H (2018) Multiple sclerosis pathology. Cold Spring Harb Perspect Med 8:a028936. 10.1101/cshperspect.a028936 29358320 10.1101/cshperspect.a028936 PMC5830904

[3] Maaloul M, Mejdoub S, Sakka S et al (2024) Infrequent patterns in cerebrospinal fluid isofocusing test: clinical significance and contribution of IgG index and Reiber diagram to their interpretation. Multiple Scler Relat Disord 84:105509. 10.1016/j.msard.2024.105509 10.1016/j.msard.2024.105509 38422634

[4] Konen FF, Wurster U, Schwenkenbecher P et al (2025) Oligoclonal bands and kappa free light chains: competing parameters or complementary biomarkers? Autoimmun Rev 24:103765. 10.1016/j.autrev.2025.103765 39947571 10.1016/j.autrev.2025.103765

[5] Comabella M, Montalban X (2014) Body fluid biomarkers in multiple sclerosis. Lancet Neurol 13:113–126. 10.1016/s1474-4422(13)70233-3 24331797 10.1016/S1474-4422(13)70233-3

[6] Khalil M, Teunissen CE, Otto M et al (2018) Neurofilaments as biomarkers in neurological disorders. Nat Rev Neurol 14:577–589. 10.1038/s41582-018-0058-z 30171200 10.1038/s41582-018-0058-z

[7] Rissin DM, Kan CW, Campbell TG et al (2010) Single-molecule enzyme-linked immunosorbent assay detects serum proteins at subfemtomolar concentrations. Nat Biotechnol 28:595–599. 10.1038/nbt.1641 20495550 10.1038/nbt.1641 PMC2919230

[8] Malmeström C, Haghighi S, Rosengren L et al (2003) Neurofilament light protein and glial fibrillary acidic protein as biological markers in MS. Neurology 61:1720–1725. 10.1212/01.wnl.0000098880.19793.b6 14694036 10.1212/01.wnl.0000098880.19793.b6

[9] Lycke JN, Karlsson J-E, Andersen O et al (1998) Neurofilament protein in cerebrospinal fluid: a potential marker of activity in multiple sclerosis. J Neurol Neurosurg Psychiatry 64:402. 10.1136/jnnp.64.3.402 9527161 10.1136/jnnp.64.3.402 PMC2170011

[10] Kuhle J, Plattner K, Bestwick JP et al (2013) A comparative study of CSF neurofilament light and heavy chain protein in MS. Multiple Scler J 19:1597–1603. 10.1177/1352458513482374 10.1177/1352458513482374 23529999

[11] Teunissen CE, Iacobaeus E, Khademi M et al (2009) Combination of CSF N-acetylaspartate and neurofilaments in multiple sclerosis. Neurology 72:1322–1329. 10.1212/wnl.0b013e3181a0fe3f 19365053 10.1212/WNL.0b013e3181a0fe3f

[12] Akgün K, Kretschmann N, Haase R et al (2019) Profiling individual clinical responses by high-frequency serum neurofilament assessment in MS. Neurol Neuroimmunol Neuroinflamm 6:e555. 10.1212/nxi.0000000000000555 31119188 10.1212/NXI.0000000000000555 PMC6501638

[13] Uher T, Schaedelin S, Srpova B et al (2020) Monitoring of radiologic disease activity by serum neurofilaments in MS. Neurol Neuroimmunol Neuroinflamm 7:e714. 10.1212/nxi.0000000000000714 32273481 10.1212/NXI.0000000000000714 PMC7176248

[14] Cantó E, Barro C, Zhao C et al (2019) Association between serum neurofilament light chain levels and long-term disease course among patients with multiple sclerosis followed up for 12 years. JAMA Neurol 76:1359–1366. 10.1001/jamaneurol.2019.2137 31403661 10.1001/jamaneurol.2019.2137 PMC6692664

[15] Kuhle J, Disanto G, Lorscheider J et al (2015) Fingolimod and CSF neurofilament light chain levels in relapsing-remitting multiple sclerosis. Neurology 84:1639–1643. 10.1212/wnl.0000000000001491 25809304 10.1212/WNL.0000000000001491 PMC4409586

[16] Novakova L, Axelsson M, Khademi M et al (2017) Cerebrospinal fluid biomarkers as a measure of disease activity and treatment efficacy in relapsing‐remitting multiple sclerosis. J Neurochem 141:296–304. 10.1111/jnc.13881 27787906 10.1111/jnc.13881

[17] Kuhle J, Kropshofer H, Haering DA et al (2019) Blood neurofilament light chain as a biomarker of MS disease activity and treatment response. Neurology. 10.1212/WNL.0000000000007032 30737333 10.1212/WNL.0000000000007032 PMC6442011

[18] Ziemssen T, Arnold DL, Alvarez E et al (2022) Prognostic value of serum neurofilament light chain for disease activity and worsening in patients with relapsing multiple sclerosis: results from the phase 3 ASCLEPIOS I and II trials. Front Immunol 13:852563. 10.3389/fimmu.2022.852563 35432382 10.3389/fimmu.2022.852563 PMC9009385

[19] Bar-Or A, Thanei G-A, Harp C et al (2023) Blood neurofilament light levels predict non-relapsing progression following anti-CD20 therapy in relapsing and primary progressive multiple sclerosis: findings from the ocrelizumab randomised, double-blind phase 3 clinical trials. EBioMedicine 93:104662. 10.1016/j.ebiom.2023.104662 37354600 10.1016/j.ebiom.2023.104662 PMC10320523

[20] Bittner S, Oh J, Havrdová EK et al (2021) The potential of serum neurofilament as biomarker for multiple sclerosis. Brain 144:2954–2963. 10.1093/brain/awab241 34180982 10.1093/brain/awab241 PMC8634125

[21] Novakova L, Zetterberg H, Sundström P et al (2017) Monitoring disease activity in multiple sclerosis using serum neurofilament light protein. Neurology 89:2230–2237. 10.1212/wnl.0000000000004683 29079686 10.1212/WNL.0000000000004683 PMC5705244

[22] Benkert P, Meier S, Schaedelin S et al (2022) Serum neurofilament light chain for individual prognostication of disease activity in people with multiple sclerosis: a retrospective modelling and validation study. Lancet Neurol 21:246–257. 10.1016/s1474-4422(22)00009-6 35182510 10.1016/S1474-4422(22)00009-6

[23] Khalil M, Teunissen CE, Lehmann S et al (2024) Neurofilaments as biomarkers in neurological disorders—towards clinical application. Nat Rev Neurol 20:269–287. 10.1038/s41582-024-00955-x 38609644 10.1038/s41582-024-00955-x

[24] Rodríguez-Jorge F, Fernández-Velasco JI, Villarrubia N et al (2024) Biomarkers of response to ocrelizumab in relapsing–remitting multiple sclerosis. Front Immunol 15:1480676. 10.3389/fimmu.2024.1480676 39606235 10.3389/fimmu.2024.1480676 PMC11600310

[25] Srpova B, Uher T, Hrnciarova T et al (2019) Serum neurofilament light chain reflects inflammation-driven neurodegeneration and predicts delayed brain volume loss in early stage of multiple sclerosis. Mult Scler J 27:52–60. 10.1177/1352458519901272 10.1177/1352458519901272 31961243

[26] Dietmann A-S, Kruse N, Stork L et al (2023) Neurofilament light chains in serum as biomarkers of axonal damage in early MS lesions: a histological–serological correlative study. J Neurol 270:1416–1429. 10.1007/s00415-022-11468-2 36372867 10.1007/s00415-022-11468-2 PMC9971126

[27] Freedman MS, Abdelhak A, Bhutani MK et al (2025) The role of serum neurofilament light (sNfL) as a biomarker in multiple sclerosis: insights from a systematic review. J Neurol 272:400. 10.1007/s00415-025-13093-1 40372550 10.1007/s00415-025-13093-1 PMC12081536

[28] Voigt I, Inojosa H, Wenk J et al (2023) Building a monitoring matrix for the management of multiple sclerosis. Autoimmun Rev 22:103358. 10.1016/j.autrev.2023.103358 37178996 10.1016/j.autrev.2023.103358

[29] Gawde S, Agasing A, Bhatt N et al (2022) Biomarker panel increases accuracy for identification of an MS relapse beyond sNfL. Mult Scler Relat Disord 63:103922. 10.1016/j.msard.2022.103922 35671674 10.1016/j.msard.2022.103922 PMC9351348

[30] Hernández FJB, Villarrubia AR, Fernández CM et al (2024) Real-world study of serum neurofilament light chain levels in ocrelizumab-treated people with relapsing multiple sclerosis. J Pers Med 14:692. 10.3390/jpm14070692 39063946 10.3390/jpm14070692 PMC11277843

[31] Sahoo S, Harper C, Tsui AKY et al (2025) Serum neurofilament light chain as a biomarker in multiple sclerosis: a cross-sectional observation in real-world clinical practice. Multiple Scler Relat Disord 103:106637. 10.1016/j.msard.2025.106637 10.1016/j.msard.2025.106637 40840335

[32] Schweitzer F, Laurent S, Cortese I et al (2023) Progressive multifocal leukoencephalopathy: pathogenesis, diagnostic tools, and potential biomarkers of response to therapy. Neurology 101:700–713. 10.1212/wnl.0000000000207622 37487750 10.1212/WNL.0000000000207622 PMC10585672

[33] Muralidharan KK, Kuesters G, Plavina T et al (2017) Population pharmacokinetics and target engagement of natalizumab in patients with multiple sclerosis. J Clin Pharmacol 57:1017–1030. 10.1002/jcph.894 28398628 10.1002/jcph.894

[34] Derfuss T, Kovarik JM, Kappos L et al (2017) α4-integrin receptor desaturation and disease activity return after natalizumab cessation. Neurol Neuroimmunol Neuroinflamm 4:e388. 10.1212/nxi.0000000000000388 28856176 10.1212/NXI.0000000000000388 PMC5572051

[35] Plavina T, Muralidharan KK, Kuesters G et al (2017) Reversibility of the effects of natalizumab on peripheral immune cell dynamics in MS patients. Neurology 89:1584–1593. 10.1212/wnl.0000000000004485 28916537 10.1212/WNL.0000000000004485 PMC5634662

[36] Ryerson LZ, Foley J, Chang I et al (2019) Risk of natalizumab-associated PML in patients with MS is reduced with extended interval dosing. Neurology. 10.1212/WNL.0000000000008243 31515290 10.1212/WNL.0000000000008243 PMC7010325

[37] Ryerson LZ, Frohman TC, Foley J et al (2016) Extended interval dosing of natalizumab in multiple sclerosis. J Neurol Neurosurg Psychiatry 87:885. 10.1136/jnnp-2015-312940 26917698 10.1136/jnnp-2015-312940

[38] van Kempen ZLE, Hoogervorst ELJ, Wattjes MP et al (2020) Personalized extended interval dosing of natalizumab in MS—a prospective multicenter trial. Neurology. 10.1212/WNL.0000000000009995 32690785 10.1212/WNL.0000000000009995

[39] Clerico M, Mercanti SFD, Signori A et al (2020) Extending the interval of natalizumab dosing: is efficacy preserved? Neurotherapeutics 17:200–207. 10.1007/s13311-019-00776-7 31452081 10.1007/s13311-019-00776-7 PMC7007494

[40] Butzkueven H, Kappos L, Spelman T et al (2021) No evidence for loss of natalizumab effectiveness with every-6-week dosing: a propensity score–matched comparison with every-4-week dosing in patients enrolled in the Tysabri Observational Program (TOP). Ther Adv Neurol Disord 14:17562864211042458. 10.1177/17562864211042458 34603507 10.1177/17562864211042458 PMC8481711

[41] Foley JF, Defer G, Ryerson LZ et al (2022) Comparison of switching to 6-week dosing of natalizumab versus continuing with 4-week dosing in patients with relapsing-remitting multiple sclerosis (NOVA): a randomised, controlled, open-label, phase 3b trial. Lancet Neurol 21:608–619. 10.1016/s1474-4422(22)00143-0 35483387 10.1016/S1474-4422(22)00143-0

[42] Bomprezzi R, Pawate S (2014) Extended interval dosing of natalizumab: a two-center, 7-year experience. Ther Adv Neurol Disord 7:227–231. 10.1177/1756285614540224 25342976 10.1177/1756285614540224 PMC4206618

[43] O’Connor PW, Goodman A, Kappos L et al (2011) Disease activity return during natalizumab treatment interruption in patients with multiple sclerosis. Neurology 76:1858–1865. 10.1212/wnl.0b013e31821e7c8a. (**e-pub ahead of print**) 21543733 10.1212/WNL.0b013e31821e7c8a

[44] Trojano M, Ramió-Torrentà L, Grimaldi LM et al (2021) A randomized study of natalizumab dosing regimens for relapsing–remitting multiple sclerosis. Mult Scler J 27:2240–2253. 10.1177/13524585211003020 10.1177/13524585211003020 PMC8597184 33821693

[45] Kaufman M, Cree BAC, Sèze JD et al (2015) Radiologic MS disease activity during natalizumab treatment interruption: findings from RESTORE. J Neurol 262:326–336. 10.1007/s00415-014-7558-6 25381458 10.1007/s00415-014-7558-6

[46] Bernardes C, Fernandes C, Cunha C et al (2024) Natalizumab extended interval dosing: what about wearing-off effect? J Neurol Sci 458:122930. 10.1016/j.jns.2024.122930 38368641 10.1016/j.jns.2024.122930

[47] Baker D, Pryce G, James LK et al (2020) The ocrelizumab phase II extension trial suggests the potential to improve the risk: benefit balance in multiple sclerosis. Mult Scler Relat Disord 44:102279. 10.1016/j.msard.2020.102279 32645640 10.1016/j.msard.2020.102279

[48] Meng D, Sacco R, Disanto G et al (2024) Memory B cell–guided extended interval dosing of ocrelizumab in multiple sclerosis. Multiple Scler J 30:857–867. 10.1177/13524585241250199 10.1177/13524585241250199 38767224

[49] Novak F, Bajwa HM, Østergaard K et al (2024) Extended interval dosing with ocrelizumab in multiple sclerosis. Multiple Scler J 30:847–856. 10.1177/13524585241245296 10.1177/13524585241245296 38646949

[50] Bisecco A, Matrone F, Capobianco M et al (2024) COVID-19 outbreak in Italy: an opportunity to evaluate extended interval dosing of ocrelizumab in MS patients. J Neurol 271:699–710. 10.1007/s00415-023-12084-4 37982852 10.1007/s00415-023-12084-4 PMC10827970

[51] Rjeily NB, Fitzgerald KC, Mowry EM (2023) Extended interval dosing of ocrelizumab in patients with multiple sclerosis is not associated with meaningful differences in disease activity. Multiple Scler J 30:257–260. 10.1177/13524585231208311 10.1177/13524585231208311 37942884

[52] Rolfes L, Pawlitzki M, Pfeuffer S et al (2021) Ocrelizumab extended interval dosing in multiple sclerosis in times of COVID-19. Neurol Neuroimmunol Neuroinflamm 8:e1035. 10.1212/nxi.0000000000001035 34261812 10.1212/NXI.0000000000001035 PMC8362352

[53] Rempe T, Elfasi A, Rodriguez E et al (2023) Ocrelizumab B-cell repopulation-guided extended interval dosing versus standard dosing—similar clinical efficacy with decreased immunoglobulin M deficiency rates. Multiple Scler Relat Disord 79:105028. 10.1016/j.msard.2023.105028 10.1016/j.msard.2023.105028 37813071

[54] Schuckmann A, Steffen F, Zipp F et al (2023) Impact of extended interval dosing of ocrelizumab on immunoglobulin levels in multiple sclerosis. Med 4:361-372.e3. 10.1016/j.medj.2023.05.001 37236189 10.1016/j.medj.2023.05.001

[55] Nasello M, Zancan V, Rinaldi V et al (2024) Clinical and immunological impact of ocrelizumab extended interval dosing in multiple sclerosis: a single-center, real-world experience. Int J Mol Sci 25:5353. 10.3390/ijms25105353 38791391 10.3390/ijms25105353 PMC11121257

[56] Thebault S, Abdoli M, Fereshtehnejad S-M et al (2020) Serum neurofilament light chain predicts long term clinical outcomes in multiple sclerosis. Sci Rep 10:10381. 10.1038/s41598-020-67504-6 32587320 10.1038/s41598-020-67504-6 PMC7316736

[57] Brummer T, Muthuraman M, Steffen F et al (2022) Improved prediction of early cognitive impairment in multiple sclerosis combining blood and imaging biomarkers. Brain Commun 4:fcac153. 10.1093/braincomms/fcac153 35813883 10.1093/braincomms/fcac153 PMC9263885

[58] Bittner S, Steffen F, Uphaus T et al (2020) Clinical implications of serum neurofilament in newly diagnosed MS patients: a longitudinal multicentre cohort study. EBioMedicine 56:102807. 10.1016/j.ebiom.2020.102807 32460167 10.1016/j.ebiom.2020.102807 PMC7251380

[59] Ashrafzadeh-Kian S, Figdore D, Larson B et al (2024) Head-to-head comparison of four plasma neurofilament light chain (NfL) immunoassays. Clin Chim Acta 561:119817. 10.1016/j.cca.2024.119817 38879065 10.1016/j.cca.2024.119817

[60] Varhaug KN, Barro C, Bjørnevik K et al (2018) Neurofilament light chain predicts disease activity in relapsing-remitting MS. Neurol Neuroimmunol Neuroinflamm. 10.1212/nxi.0000000000000422 29209636 10.1212/NXI.0000000000000422 PMC5707445

[61] Disanto G, Barro C, Benkert P et al (2017) Serum neurofilament light: a biomarker of neuronal damage in multiple sclerosis. Ann Neurol 81:857–870. 10.1002/ana.24954 28512753 10.1002/ana.24954 PMC5519945

[62] Baker D, Pryce G, Amor S et al (2018) Learning from other autoimmunities to understand targeting of B cells to control multiple sclerosis. Brain 141:2834–2847. 10.1093/brain/awy239 30212896 10.1093/brain/awy239

[63] Zecca C, Bovis F, Novi G et al (2019) Treatment of multiple sclerosis with rituximab: a multicentric Italian–Swiss experience. Multiple Scler J 26:1519–1531. 10.1177/1352458519872889 10.1177/1352458519872889 31573386

[64] Wiendl H, Gold R, Berger T et al (2021) Multiple Sclerosis Therapy Consensus Group (MSTCG): position statement on disease-modifying therapies for multiple sclerosis (white paper). Ther Adv Neurol Disord 14:17562864211039648. 10.1177/17562864211039648 34422112 10.1177/17562864211039648 PMC8377320

[65] Toorop AA, van Lierop ZY, Gelissen LM et al (2024) Prospective trial of natalizumab personalised extended interval dosing by therapeutic drug monitoring in relapsing-remitting multiple sclerosis (NEXT-MS). J Neurol Neurosurg Psychiatry 95:392–400. 10.1136/jnnp-2023-332119 37963723 10.1136/jnnp-2023-332119

[66] Ruggieri S, Ianniello A, Copetti M et al (2024) Treatment modifiers across different regimens of natalizumab treatment in MS: an Italian real-world experience. Neurotherapeutics 21:e00338. 10.1016/j.neurot.2024.e00338 38413275 10.1016/j.neurot.2024.e00338 PMC11070710

[67] Gibiansky E, Petry C, Mercier F et al (2021) Ocrelizumab in relapsing and primary progressive multiple sclerosis: pharmacokinetic and pharmacodynamic analyses of OPERA I, OPERA II and ORATORIO. Br J Clin Pharmacol 87:2511–2520. 10.1111/bcp.14658 33202059 10.1111/bcp.14658 PMC8247316

