# Supplementary material for: Serum neurofilament light chain as a biomarker of disease control in multiple sclerosis: a real-world cross-sectional analysis of therapeutic regimens
Source: J Neurol. 2025 Dec 19;273(1):34. doi: 10.1007/s00415-025-13573-4 (PMC12717141; doi:10.1007/s00415-025-13573-4)
Supplement: Supplementary file 1 — Supplementary file1 (DOCX 162 KB) [file 415_2025_13573_MOESM1_ESM.docx]

**Supplementary Table 1.** A. ANCOVA results for the overall model when comparing sNfL levels between RRMS and PPMS/SPMS. B. Post-hoc analysis of sNfL differences between RRMS and PPMS/SPMS.

| 1. **ANCOVA** | |  |  |  |  |
| --- | --- | --- | --- | --- | --- |
|  | |  |  | **p** |  |
| **Leven's Test of Equality of Error Variances** | |  |  | 0.679 |  |
|  | | | | | |
| **Variable** | | **Mean square** | **F** | **p** | **Partial eta squared** |
| MS course (RRMS vs. PPMS/SPMS) | | 0.233 | 0.552 | 0.577 | 0.007 |
| **Covariate** | | **Mean square** | **F** | **p** | **Partial eta squared** |
| Age | | 31.874 | 75.42 | <0.001 | 0.322 |
| 1. **Post-hoc analysis** |  |  | |  | |
|  |  | **95% CI** | |  | |
| **MS course** | **Mean estimated sNfL (pg/ml)** | lower | upper | **p** | |
| RRMS | 1.517 | 1.404 | 1.630 | 0.6577 | |
| PPMS | 1.473 | 1.129 | 1.817 |  |  |
| SPMS | 1.688 | 1.364 | 2.013 |  |  |

**Legend Supplementary Table 1:** ANCOVA: Analysis of covariance, CI: confidence interval, sNfL: serum neurofilament light chain (protein), RRMS: relapsing-remitting MS, SPMS: secondary progressive MS, PPMS: primary progressive MS

**Supplementary Figure 1.** Histograms illustrating the distribution of sNfL values before and after log₁₀ transformation.

**
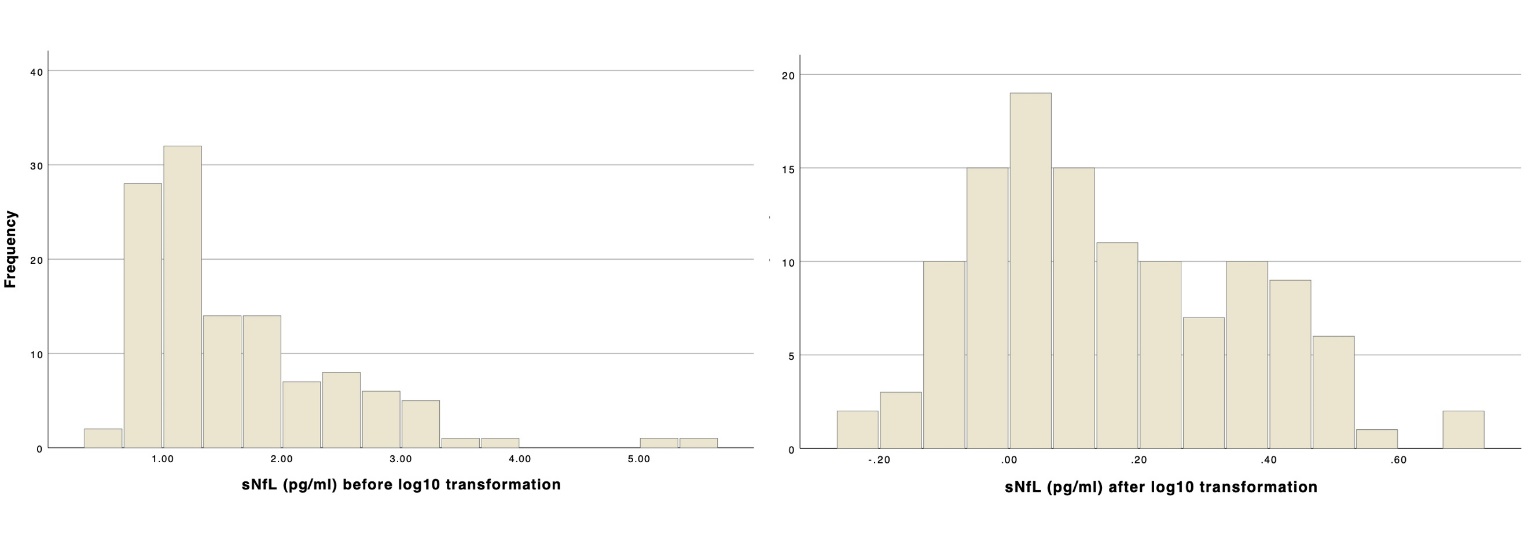
**

**Legend Supplementary Figure 1:** sNfL: serum neurofilament light chain (protein)
